# Low prevalence of laboratory-confirmed malaria in clinically diagnosed adult women from the Wakiso district of Uganda

**DOI:** 10.1186/s12936-016-1604-z

**Published:** 2016-11-14

**Authors:** Sergey Yegorov, Ronald M. Galiwango, Aloysious Ssemaganda, Moses Muwanga, Irene Wesonga, George Miiro, David A. Drajole, Kevin C. Kain, Noah Kiwanuka, Bernard S. Bagaya, Rupert Kaul

**Affiliations:** 1 Departments of Medicine and Immunology, University of Toronto, 1 King’s College Circle, 6356, Toronto, ON M5S1A8 Canada; 2 UVRI-IAVI HIV Vaccine Program, Plot 50-55 Nakiwogo Road, Entebbe, Uganda; 3 Institute for Glycomics, Griffith University, Gold Coast, Parklands Drive, Southport, QLD 4215 Australia; 4 Entebbe General Hospital, P.O. Box 29, Entebbe, Uganda; 5 Uganda Virus Research Institute, Plot 51-59 Nakiwogo Road, Entebbe, Uganda; 6 Sandra A. Rotman Laboratories, Sandra Rotman Centre for Global Health, MaRS Centre, University Health Network, 101 College St. TMDT 10-360A, Toronto, ON M5G1L7 Canada; 7 Tropical Disease Unit, Division of Infectious Diseases, Department of Medicine, University of Toronto, Toronto, Canada; 8 Department of Epidemiology and Biostatistics, School of Public Health, College of Health Sciences, Makerere University, P.O. Box 7072, Kampala, Uganda; 9 Department of Immunology and Molecular Biology, School of Biomedical Sciences, College of Health Sciences, Makerere University, P.O. Box 7072, Kampala, Uganda

**Keywords:** Adult malaria management, Malaria overdiagnosis, Malaria overtreatment, Microscopy, RDT, PCR

## Abstract

**Background:**

The malaria burden in sub-Saharan Africa (SSA) has fallen substantially. Nevertheless, malaria remains a serious health concern, and Uganda ranks third in SSA in total malaria burden. Epidemiological studies of adult malaria in Uganda are scarce and little is known about rates of malaria in non-pregnant adult women. This pilot study assessed malaria prevalence among adult women from Wakiso district, historically a highly malaria endemic region.

**Methods:**

Adult women using public health services were screened for malaria, HIV and pregnancy. A physician-selected subset of women presenting to the Outpatient Department of Entebbe General Hospital (EGH) with current fever (axillary temperature ≥37.5 °C) or self-reporting fever during the previous 24 h, and a positive thick smear for malaria in the EGH laboratory were enrolled (n = 86). Women who self-identified as pregnant or HIV-positive were excluded from screening. Malaria infection was then assessed using HRP2/pLDH rapid diagnostic tests (RDTs) in all participants. Repeat microscopy and PCR were performed at a research laboratory for a subset of participants. In addition, 104 women without a history of fever were assessed for asymptomatic parasitaemia using RDT, and a subset of these women screened for parasitaemia using microscopy (40 women) and PCR (40 women).

**Results:**

Of 86 women diagnosed with malaria by EGH, only two (2.3%) had malaria confirmed using RDT, subsequently identified as a *Plasmodium falciparum* infection by research microscopy and PCR. Subset analysis of hospital diagnosed RDT-negative participants detected one sub-microscopic infection with *Plasmodium ovale*. Compared to RDT, sensitivity, specificity and PPV of hospital microscopy were 100% (CI 19.8–100), 0% (CI 0–5.32) and 2.33% (CI 0.403–8.94) respectively. Compared to PCR, sensitivity, specificity and PPV of hospital microscopy were 100% (CI 31.0–100), 0% (CI 0–34.5) and 23.1% (CI 6.16–54.0), respectively. No malaria was detected among asymptomatic women using RDT, research microscopy or PCR.

**Conclusions:**

Malaria prevalence among adult women appears to be low in Wakiso, but is masked by high rates of malaria overdiagnosis. More accurate malaria testing is urgently needed in public hospitals in this region to identify true causes of febrile illness and reduce unnecessary provision of anti-malarial therapy.

## Background

Although great progress has been made over the past one and a half decades in reducing the burden of malaria [1], the disease remains a leading cause of morbidity and mortality in sub-Saharan Africa (SSA). Because young children bear the brunt of malaria-associated morbidity, an understanding of the malaria burden in adults is fragmented and assessment of malaria control in this population is needed [2–4]. Uganda ranked fourth globally and third in SSA in terms of the total malaria cases in 2015 [5], but most epidemiological data until recently were obtained from surveys in young children. The 2014–2015 Malaria Indicator Survey, for instance, reported that the average prevalence of malaria was 30% (varying from 3.7 to 51.3% based on the region) in Ugandan children aged 0–59 months as assessed using malaria rapid diagnostic tests (RDT) and 19% (0.4–36.2%) as assessed by light microscopy (LM) in the same age cohort [6]. A 2011–2013 community-based survey conducted in three Ugandan sub-counties with varied transmission settings performed the first rigorous assessment of malaria metrics in both children and adults [7–9]. According to the results of this survey, malaria prevalence based on LM ranged from 3.0 to 5.1% among adults aged ≥18, while prevalence based on both LM and molecular techniques ranged from 18.8 to 53.5% in the same age group [8]. This is one of the first detailed studies on the prevalence of malaria in adults, but additional data from other regions and broad age groups is needed to adequately monitor malaria trends in Uganda [10].

Sub-Saharan Africa health facilities tend to over-diagnose malaria in patients presenting with symptoms such as fever, due to traditional perceptions (e.g. perceptions of high malaria endemicity and any fever being equivalent to malaria) and issues related to laboratory testing [11–14]. The gold standard for malaria diagnosis is microscopic examination of blood smears. However, maintaining high standards of LM requires multiple pre-requisites, which are difficult to maintain in resource-limited settings in SSA [15]. To aid parasite-based malaria diagnosis, RDTs have recently been incorporated into clinical guidelines of malaria-endemic countries [5]. RDTs that detect circulating Plasmodium antigens, histidine-rich protein 2 (HRP2) and lactate dehydrogenase (pLDH), perform similarly well to expert LM, and can often exceed the quality of field LM in clinical studies [11, 16–19]. Importantly, RDTs require minimal training and equipment, hence making them a feasible malaria-testing tool for primary care workers with limited laboratory experience.

The objective of this pilot study was to assess malaria prevalence among adult women with and without a history of fever, who were accessing public health services in Entebbe, Wakiso district, Uganda. Historically, Entebbe has been classified as a malaria hyper-endemic area [20, 21] with an estimated malaria prevalence of 13% in young children [6]. Most studies of malaria rates in adults in the region have focused on pregnant women or HIV-infected persons [22–24] and as a result the effectiveness of recent interventions in reducing malaria prevalence in the general population is unknown. An HRP2/pLDH RDT was used to screen participants for malaria and to confirm hospital LM-based diagnosis.

## Methods

### Study site

**Fig. 1:**
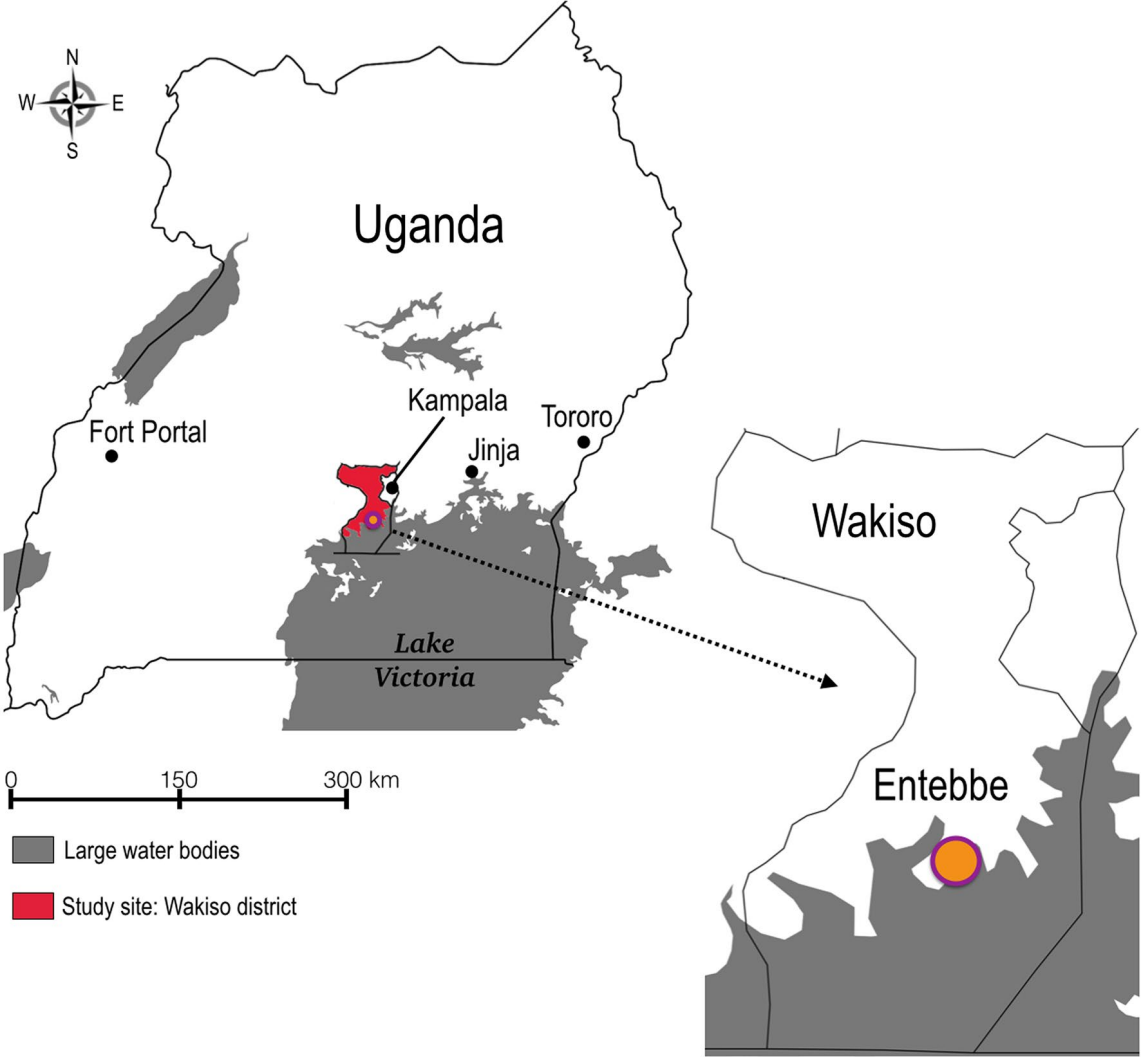
Map of Uganda showing the location of the study site (Entebbe, Wakiso District). The map was created using an online tool SimpleMappr [40]

Entebbe is located in Central Uganda (Fig. 1). This region is a peninsula in Lake Victoria inhabited by semi-urban, rural and fishing communities. Rainfall patterns in Entebbe are bimodal with rainy seasons in March–May and in September–November, and peak malaria transmission tends to occur several weeks after the end of the rainy season [6, 20]. Malaria infection in this region is caused primarily by *Plasmodium falciparum*, but infection by *Plasmodium malariae* and *Plasmodium ovale* has also been observed [6]. Entebbe General Hospital (EGH) Outpatient Department (OPD) offers government-subsidized services to the residents of Entebbe town and of the peri-urban communities surrounding Entebbe.

### Participant characteristics

**Fig. 2:**
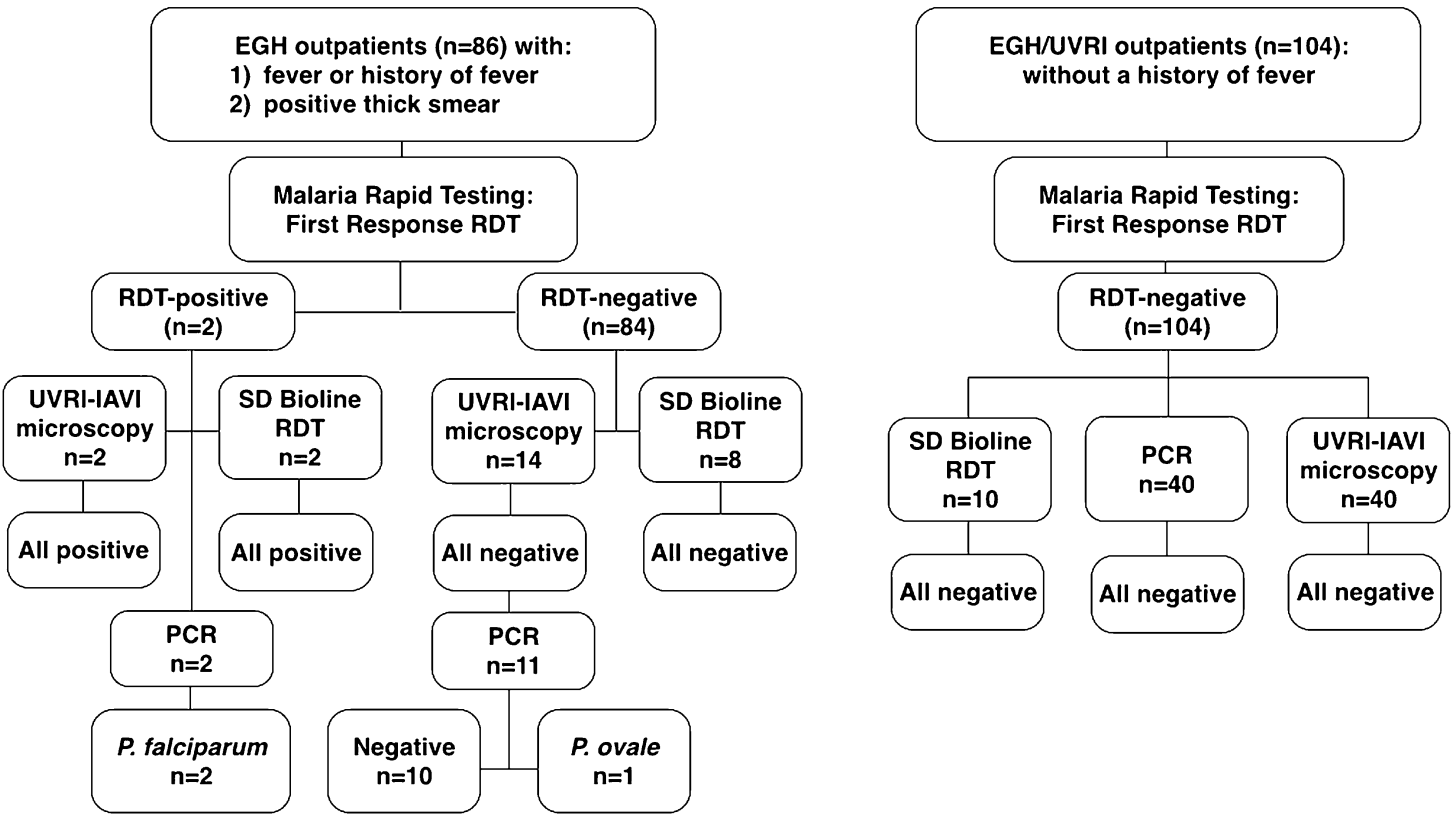
*Left panel* diagnostic algorithm for adult women with suspected malaria at the outpatient department (OPD) of Entebbe General Hospital (EGH). *Right panel* diagnostic algorithm for adult afebrile women attending outpatient clinics at EGH and the Uganda Virus Research Institute (UVRI). *RDT* malaria rapid diagnostic test

As a precursor to studies on the immune impact of malaria infection, women aged 18-45 years were screened for malaria, HIV and pregnancy between July-December 2015 (see below and Table 1). A physician-selected subset of women presenting to the EGH OPD with fever (axillary temperature ≥37.5 °C) or a self-report of fever during the previous 24 h, and a positive thick smear for malaria in the EGH laboratory were enrolled (n = 86; Fig. 2; Table 1). Women who self-identified as pregnant or HIV-positive were excluded from screening.

**Table 1:** Clinical characteristics of study participants

Parameter	Fever group	No fever group
Age: median (range)	25 (18–45)	29 (18–45)
RDT-positive	2.3 (2/86)	0 (0/104)
HIV-positive %	2.3 (2/86)	4.8 (5/104)
Pregnant %	20.9 (18/86)	3.8 (4/104)

Malaria LM is one of the most frequently requested laboratory tests at EGH. During the study period, the EGH laboratory staff rotated on a weekly basis and a total of three technicians performed blood smear analysis. On any given day, only one microscopist was at the LM bench. Individuals diagnosed with malaria by the EGH clinician received artemisinin-based combination therapy (ACT) and/or quinine according to the Uganda clinical guidelines. HIV rapid testing was conducted using the Uganda Ministry of Health testing algorithm [25]. Pregnancy was tested using QuickVue One-Step hCG urine test (Quidel Corporation, USA).

### RDT screening

Malaria infection was then assessed using RDT in all participants. At all sites, two RDTs meeting the WHO performance criteria [26] were employed to detect a *P. falciparum* or mixed *Plasmodium* species infection. The First Response Malaria Ag pLDH/HRP2 (Premier Medical Corp Ltd, India), validated by previous studies from Uganda, India and Yemen to have high performance characteristics [19, 27, 28], was used on all participants, and the SD Bioline Malaria Ag Pf/Pan (Standard Diagnostics Inc, South Korea), previously validated for routine use at the UVRI-IAVI HIV Vaccine program, was also used to retest 10% of samples for quality control. RDT kits were stored at room temperature as recommended by the manufacturer. Study personnel were trained in the safe use and interpretation of the RDT following the manufacturer’s instructions. Approximately 5 μl of capillary or venous blood was tested and results were read and interpreted within 20 min. RDT results were considered valid only if the control test line was positive.

### Repeat LM and PCR testing

Confirmatory thick and thin smear LM analysis was performed for a subset of participants (n = 16) at the UVRI-IAVI HIV Vaccine Programme’s research laboratory as follows. Blood was transported to the research laboratory, where two blinded microscopists prepared thick and thin blood films using Field stain and separately performed LM. A blood film was considered negative if no parasites were detected after 100 high power fields had been examined. If parasites were observed, counting was performed against 200 white blood cells on the thick film. The thin film was used for malaria species identification and known positive/negative samples were used as test controls. Discrepant LM results were resolved by the UVRI Medical Research Council Clinical Diagnostics Lab Services (MRC/UVRI CDLS). The UVRI-IAVI research microscopists receive extended practical training in malaria parasitology and LM at MRC/UVRI CDLS and participate in quarterly inter-laboratory comparisons with the MRC/UVRI CDLS experts. In the last three years, the UVRI-IAVI research laboratory has also participated with satisfactory performance in the Royal College of Pathologists of Australasia (RCPA)-Malaria External Quality Assurance Programme.

For PCR investigation, dried blood spots (DBS) were prepared by blotting blood onto Whatman FTA cards (GE Healthcare, UK) kept at room temperature until DNA extraction. Nested *Plasmodium* spp.-specific PCR was then performed at the University of Toronto, as described elsewhere [29] on thirteen participants that had a repeat LM result.

### Screening for asymptomatic malaria

To assess malaria prevalence among asymptomatic individuals, women without a history of fever (n = 104) who were attending the family planning or child vaccination clinics at either the Mother and Child Health (MCH) department of EGH or the Uganda Virus Research Institute (UVRI) outpatient clinic were enrolled for malaria screening using RDT. In addition, a randomly chosen set of 40 women underwent thick and thin smear LM at UVRI-IAVI and another set of 40 participants was tested using nested PCR.

## Results

Among 86 OPD women with fever or a history of fever and a positive blood thick smear as reported by the EGH laboratory, approximately 20% were pregnant and 2% were HIV-infected (Table 1). Only 2.3% (2/86) were positive on the RDT; these women were 24 and 30 years old and were not pregnant or HIV-infected. Of the 84 women with a clinical diagnosis of malaria but a negative RDT, confirmatory LM testing was performed for 14 participants, as well as for both RDT-positive participants (Fig. 2). Additionally, PCR was performed on a subset of thirteen samples (both RDT-positive and nine RDT-negative samples). RDT-positive samples were confirmed by both research (UVRI-IAVI) LM and by PCR as *P. falciparum* positive. Out of fourteen participants that had been initially scored positive by the EGH laboratory, none were scored positive by research LM. In a subset of eleven RDT-negative participants that were also scored negative by research LM, one sample was positive for *P. ovale* by PCR (Fig. 2). Performance characteristics of hospital LM and RDT are summarized in Table 2. Compared to RDT, sensitivity, specificity and PPV of hospital LM were 100% (CI 19.8–100), 0% (CI 0–5.32) and 2.33% (CI 0.403–8.94) respectively. Compared to research LM, sensitivity, specificity and PPV of hospital LM were 100% (CI 19.8–100), 0% (CI 0–26.8) and 12.5% (CI 2.20–39.59), respectively. Compared to PCR, sensitivity, specificity and PPV of hospital LM were 100% (CI 31.0–100), 0% (CI 0–34.5) and 23.1% (CI 6.16–54.0), respectively. Sensitivity, specificity and PPV of RDT compared to research LM were 100% (CI 19.8–100), 100% (CI 73.2–100) and 100% (CI 19.8–100), respectively. Compared to PCR, sensitivity, specificity and PPV of RDT were 66.7% (CI 12.5–98.2), 100% (CI 65.5–100) and 100% (CI 19.8–100), respectively.

**Table 2:** Performance of hospital LM (compared to RDT, research LM and PCR) and RDT (compared to PCR)

Diagnostic comparison	True+	True−	False+	False−	Sensitivity (95% CI)	Specificity (95% CI)	PPV (95% CI)
EGH LM vs. RDT	2	0^a^	84	0	100.0 (19.8–100)	0 (0–5.32)^a^	2.33 (0.403–8.94)
EGH LM vs. research LM	2	0^a^	14	0	100.0 (19.8–100)	0 (0–26.8)^a^	12.5 (2.20–39.59)
EGH LM vs. PCR	3	0^a^	10	0	100.0 (31.0–100)	0 (0–34.5)^a^	23.1 (6.16–54.0)
RDT vs. research LM	2	14	0	0	100.0 (19.8–100)	100 (73.2–100)	100 (19.8–100)
RDT vs. PCR	2	10	0	1	66.7 (12.5–98.2)	100 (65.5–100.0)	100 (19.8–100)

^a^Only malaria–diagnosed subjects were included in the study, hence the true estimate for this value is unknown. *PPV* positive predictive value

The prevalence of HIV in the asymptomatic group of 104 women was 4.8% and 3.8% were pregnant (Table 1). No malaria was detected among these women using RDT, and in two randomly chosen subsets by research LM (40 individuals) or PCR (40 individuals) (Fig. 2).

## Discussion

Overall, this study found high rates of malaria overdiagnosis and overtreatment at a major public health facility in Uganda. Most blood smears reported as positive by the hospital laboratory were not confirmed using RDT (97%) nor in a smaller sample subset, by research LM (88%) or PCR (77%). Overall, hospital LM had high sensitivity (100%), but very low specificity and positive predictive values compared to RDT, research LM and PCR (Table 2). This reinforces well-described difficulties in the LM-based diagnosis of malaria in the public healthcare sector [11, 15, 30]. RDTs have been proposed as a feasible and more accurate alternative to LM in resource-limited settings [11, 16, 17] and multiple SSA studies report reduction in antimalarial drug prescription after RDT implementation [18, 31, 32], further strengthening the incentive for RDT use in clinical practice. However, RDTs had been under-utilized by the EGH laboratory personnel due to irregular supply and lack of confidence in RDT-based results. Based on the findings presented here, a more rigorous quality control programme is already being implemented for blood smear LM at the EGH laboratory, and the regular use of RDT in routine practice is being mandated.

Importantly, a very high false-positive rate of malaria diagnosis would be expected to lead to substantial malaria overtreatment, with the ensuing possibility of adverse drug effects, as well as under-management of other potentially important clinical causes of fever in the OPD. In agreement with the latter, SSA clinical studies found a higher case fatality rate among hospital patients misdiagnosed with malaria, compared to true malaria cases [12]. In addition, over-prescription of malaria drugs has implications for the emergence of parasite drug resistance, an important global concern [33]. Despite all this, malaria overdiagnosis remains a well-documented yet persistent issue in SSA resulting in over-inflation of actual malaria rates reported at the local and national levels [11–14].

Recent data regarding adult malaria prevalence rates in SSA and Uganda are limited [3, 4], as most malaria surveys focus on the infection in children (e.g., [6, 9, 34]). In 2008, a survey performed in the Tororo district of eastern Uganda found a malaria prevalence of 10–20% among adult women, with the highest prevalence in younger women [35]. On the other hand, data extracted from the UVRI Clinic’s malaria testing records between 2014 and 2016 show RDT positivity rates for febrile adult women from Entebbe of 6.3% (61/975), and the rate for the period overlapping the study reported here (July–December 2015) was 6.2% (13/211; Miiro G and Drajole, D A, pers comm). These lower rates are more similar to the rates reported here, and may reflect geographic variation as well as the impact of local bed net programmes, rapid urbanization [34] and education about malaria prevention practices [36]. Interestingly, RDT positivity rates reported by the UVRI clinic for men aged 18–45 were higher than in women [2014–2016 rate = 12% (74/628), July–December 2015 = 8.5% (9/106)], reflecting gender-specific differences in malaria rates previously observed in other SSA studies [3, 35].

In the most recent survey from three sub-counties (Walukuba and Nagongera, both in Jinja district, Eastern Uganda and Kihini in Kanungu district, Southwestern Uganda) LM-based rates among adults peaked at 5.0%. The survey also uncovered six to tenfold higher rates of parasitaemia using a combination of LM and molecular techniques, highlighting high levels of previously unappreciated sub-microscopic parasitaemia [8]. In this study asymptomatic women did not have parasitaemia detectable by RDT, LM or PCR, implying that malaria rates may be low in the population. However this should be investigated further in a larger sample of symptomatic and asymptomatic adults.

The RDT used in this study had high sensitivity and specificity compared to research LM, but lower sensitivity compared to PCR (Table 2), which detected *P. ovale* DNA in a sample scored negative by both research LM and RDT. This highlights an important limitation of RDTs- their low sensitivity for detection of *P. ovale* and *P. malariae* infections [37], which are implicated in ≈9.1% of malaria cases identified in the Central region of Uganda [6]. It is plausible that up to 9% of the febrile outpatients had patent *P. ovale* or *P. malariae* infections that were identified by the hospital microscopists but were not flagged by the RDT. Interestingly, the *P. ovale* PCR-positive individual was scored negative by research LM, implying that the patient had sub-microscopic parasitaemia. Sub-microscopic malaria is often not associated with clinical symptoms [9, 38], and hence was not a likely cause of fever in the majority of malaria-diagnosed RDT-negative outpatients.

The findings presented here have limitations. First, this study was designed as a pilot to explore the prevalence of malaria among febrile outpatients and was intended neither to be a true malaria survey of the population nor a rigorous comparison of different malaria diagnostics. Rather, the study relied primarily on a previously validated RDT for malaria screening. The RDT’s validated high sensitivity for *P. falciparum* ensured detection of a majority of symptomatic malaria cases, however a minority of cases consisting of *P. ovale* and *P. malariae* would have been missed by the test. RDTs also tend to be less reliable at low parasite density settings, where PCR testing is recommended to confirm RDT performance [37]. However, DBS samples were not available from all study participants to assess the prevalence of sub-patent malaria by PCR among the febrile outpatients. Currently there are no standardized methods available for direct quality control of RDT performance in the field. Therefore indirect methods, such as another RDT (SD Bioline), LM and PCR were used in this study to confirm the First Response RDT results. It was not possible to establish the impact each hospital microscopist had on the overall performance of EGH microscopy, which probably varied depending on individual expertise [30]. Data were not collected on recent self-medication with antimalarial drugs, which would also have impacted the diagnostic accuracy of malaria tests. Lastly, since up to 80% of febrile individuals in Uganda seek treatment outside public health facilities [39], recruitment of febrile women through the OPD could have led to under-estimation of malaria as a cause for febrile illness in the district. Intriguingly, a larger proportion of febrile RDT-negative women (20.9%) had unsuspected pregnancy compared to asymptomatic women by HCG test (3.8%, Table 1). The implication of this finding is unclear and should be investigated in future studies.

## Conclusions

In summary, a lower than anticipated prevalence of malaria was detected among adult women in Entebbe, Wakiso district and high rates of malaria overdiagnosis by LM were observed in a local public health facility. This confirms previously described difficulties in the LM-based diagnosis of malaria, and emphasizes the importance of regular quality control and RDT use to avoid misdiagnosis and mistreatment of fever. These findings may not be uncommon in other regions of Uganda and SSA, where successful reductions in malaria transmission may be masked by sub-optimal diagnostic practices leading to over-inflation of perceived malaria burden. In many regions, malaria in pregnant women and children remains a major public health problem and more efficient allocation of resources for treatment and eradication of this infection may be urgently needed. This pilot study provides background for future more detailed investigations of malaria dynamics in Wakiso.
